# Pre-dosing with lilotomab prior to therapy with ^177^Lu-lilotomab satetraxetan significantly increases the ratio of tumor to red marrow absorbed dose in non-Hodgkin lymphoma patients

**DOI:** 10.1007/s00259-018-3964-9

**Published:** 2018-02-22

**Authors:** Caroline Stokke, Johan Blakkisrud, Ayca Løndalen, Jostein Dahle, Anne C. T. Martinsen, Harald Holte, Arne Kolstad

**Affiliations:** 1 Department of Diagnostic Physics, Division of Radiology and Nuclear Medicine, Oslo University Hospital, Oslo, Norway; 2 Department of Life Sciences and Health, Oslo Metropolitan University, Oslo, Norway; 3 Division of Radiology and Nuclear Medicine, Oslo University Hospital, Oslo, Norway; 4 Nordic Nanovector ASA, Oslo, Norway; 5 The Department of Physics, University of Oslo, Oslo, Norway; 6 Department of Oncology, Radiumhospitalet, Oslo University Hospital, Oslo, Norway

**Keywords:** Non-Hodgkin lymphoma, Antibody radionuclide conjugate therapy, Radioimmunotherapy, Molecular radiotherapy, Dosimetry, ^177^Lu-lilotomab satetraxetan

## Abstract

**Purpose:**

^177^Lu-lilotomab satetraxetan is a novel anti-CD37 antibody radionuclide conjugate for the treatment of non-Hodgkin lymphoma (NHL). Four arms with different combinations of pre-dosing and pre-treatment have been investigated in a first-in-human phase 1/2a study for relapsed CD37+ indolent NHL. The aim of this work was to determine the tumor and normal tissue absorbed doses for all four arms, and investigate possible variations in the ratios of tumor to organs-at-risk absorbed doses.

**Methods:**

Two of the phase 1 arms included cold lilotomab pre-dosing (arm 1 and 4; 40 mg fixed and 100 mg/m^2^ BSA dosage, respectively) and two did not (arms 2 and 3). All patients were pre-treated with different regimens of rituximab. The patients received either 10, 15, or 20 MBq ^177^Lu-lilotomab satetraxetan per kg body weight. Nineteen patients were included for dosimetry, and a total of 47 lesions were included. The absorbed doses were calculated from multiple SPECT/CT-images and normalized by administered activity for each patient. Two-sided Student’s* t* tests were used for all statistical analyses.

**Results:**

Organs with distinct uptake of ^177^Lu-lilotomab satetraxetan, in addition to tumors, were red marrow (RM), liver, spleen, and kidneys. The mean RM absorbed doses were 0.94, 1.55, 1.44, and 0.89 mGy/MBq for arms 1–4, respectively. For the patients not pre-dosed with lilotomab (arms 2 and 3 combined) the mean RM absorbed dose was 1.48 mGy/MBq, which was significantly higher than for both arm 1 (*p* = 0.04) and arm 4 (*p* = 0.02). Of the other organs, the highest uptake was found in the spleen, and there was a significantly lower spleen absorbed dose for arm-4 patients than for the patient group without lilotomab pre-dosing (1.13 vs. 3.20 mGy/MBq; *p* < 0.01).

Mean tumor absorbed doses were 2.15, 2.31, 1.33, and 2.67 mGy/MBq for arms 1–4, respectively. After averaging the tumor absorbed dose for each patient, the patient mean tumor absorbed dose to RM absorbed dose ratios were obtained, given mean values of 1.07 for the patient group not pre-dosed with lilotomab, of 2.16 for arm 1, and of 4.62 for arm 4. The ratios were significantly higher in both arms 1 and 4 compared to the group without pre-dosing (*p* = 0.05 and *p* = 0.02). No statistically significant difference between arms 1 and 4 was found.

**Conclusions:**

RM is the primary dose-limiting organ for ^177^Lu-lilotomab satetraxetan treatment, and pre-dosing with lilotomab has a mitigating effect on RM absorbed dose. Increasing the amount of lilotomab from 40 mg to 100 mg/m^2^ was found to slightly decrease the RM absorbed dose and increase the ratio of tumor to RM absorbed dose. Still, both pre-dosing amounts resulted in significantly higher tumor to RM absorbed dose ratios. The findings encourage continued use of pre-dosing with lilotomab.

**Electronic supplementary material:**

The online version of this article (10.1007/s00259-018-3964-9) contains supplementary material, which is available to authorized users.

## Introduction

Antibody-radionuclide-conjugates (ARCs) based on CD20 antibodies have been used routinely for the treatment of non-Hodgkin lymphoma (NHL), and two ARCs are currently FDA-approved; ^131^iodine-tositumomab (Bexxar) and ^90^yttrium-ibritumomab tiuxetan (Zevalin) [1]. ^177^Lu-lilotomab satetraxetan or Betalutin® (Nordic Nanovector ASA, Oslo, Norway) is a novel ARC that targets the internalizing CD37 antigen, which is expressed on normal and malignant B-cells [2, 3]. During B-cell development, the CD37 antigen is found on mature B-cells, but it is absent on plasma cells and normal stem cells [4, 5]. The ARC therapy is currently under investigation in the phase 1/2a LYMRIT-37-01 trial for patients with relapsed CD37+ B-cell NHL. Four different combinations of pre-dosing and pre-treatment have been investigated in the phase 1 study. Two arms with “cold” lilotomab antibody pre-dosing of 40 mg fixed dosage (arm 1) and 100 mg/m^2^ body surface area (BSA) dosage (arm 4), and two without (arms 2 and 3). In addition, all patients were pre-treated with different regimens of rituximab, which targets the CD20 antigen, before the ^177^Lu-lilotomab satetraxetan injection. Investigating arms 1 and 2, we have previously shown that red bone marrow (RM) is the primary dose-limiting organ for the treatment, and that hematological toxicity was more severe for patients receiving higher RM doses [6]. RM absorbed doses were lower in arm 1 vs. arm 2. Tumor absorbed doses have been previously reported for ^177^Lu-lilotomab satetraxetan patients, without revealing any significant differences between the first two arms [7]. Theoretically, the absorbed dose for a given tissue can be increased or decreased by adjusting the amount of radioactivity prescribed to a patient; however, the absorbed doses for all other tissues will be shifted by the same factor. The ratio of tumor to organs-at-risk absorbed doses is therefore a parameter of vital interest when determining the pre-dosage and pre-treatment regimen that optimizes the biodistribution.

The aim of this work was to use the SPECT/CT data to determine tumor and normal tissue absorbed doses for ^177^Lu-lilotomab satetraxetan patients in all four arms of the phase 1 trial. Furthermore, potential variations in biodistribution and ratios of tumor to RM absorbed doses were to be determined.

## Materials and methods

### Patient characteristics

**Fig. 1:**
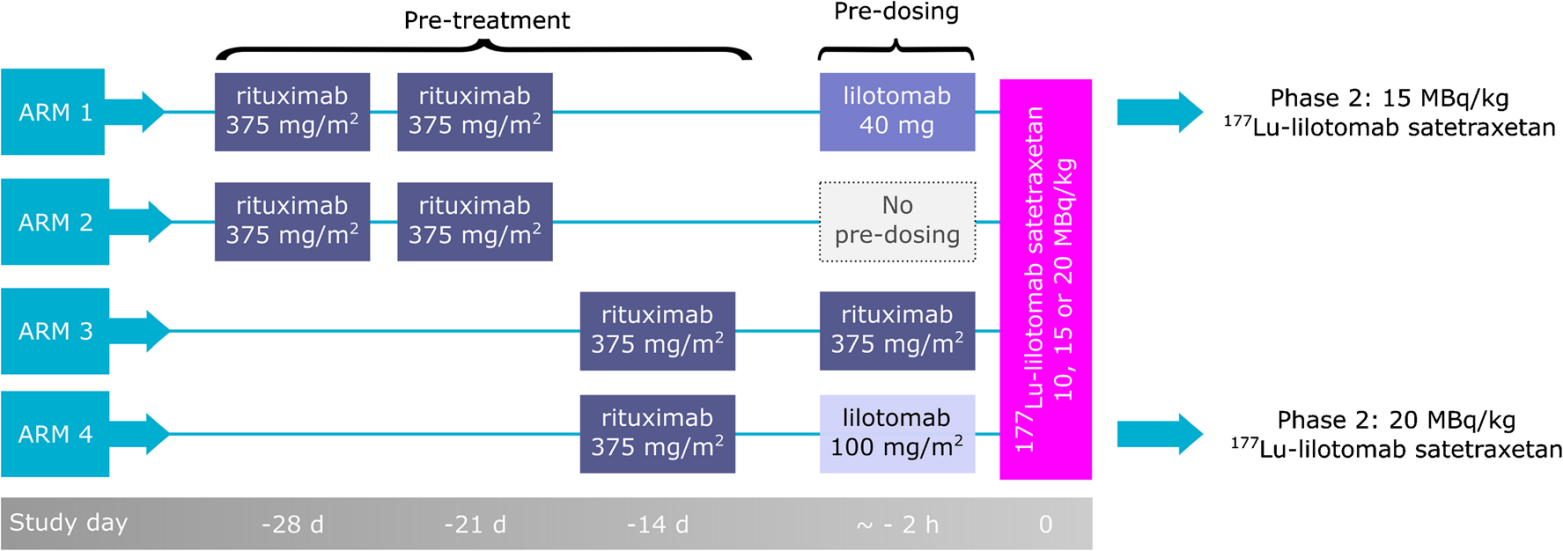
Study design of the four arms in the phase 1/2a trial. Different pre-dosing (given approximately 1–3 h before ^177^Lu-lilotomab satetraxetan injection) and pre-treatment regimens are shown in parallel. The anti-CD20 antibody rituximab was given to all patients, while only arms 1 and 4 patients received cold lilotomab (anti-CD37 antibody). The zero-hour timepoint of the grey timeline is set according to the administration of ^177^Lu-lilotomab satetraxetan. Two of the arms, 1 and 4, are continued in phase 2 as indicated

A total of 19 patients with relapsed indolent B-cell non-Hodgkin lymphoma treated at Oslo University Hospital were included in the dosimetry study. Of these 19 patients, subtypes included follicular grades I–II (16 patients), mantle cell (two patients), and marginal zone (one patient). The CD37 status of the patients was histologically confirmed. The phase 1/2a trial was approved by the regional ethical committee and all patients gave written consent. In the phase 1 trial, patients received a fixed amount of ^177^Lu-lilotomab satetraxetan radioactivity per total body mass; 10, 15, or 20 MBq/kg (Table 1). Approximately 4–10 mg of radiolabeled antibody is injected in a typical patient (75 kg body mass, 15 MBq/kg), and the mean amount was 8 mg for the patients included here. The specific activity ranged from 94 to 347 MBq/mg (mean, 186 MBq/mg). Individual patient characteristics can be found in Suppl. Table 1. Four treatment arms were investigated (Fig. 1). The infusion of lilotomab took approximately 1 h for the patients, and the indicated lilotomab pre-dosing injection was finished on average 1.7 h (range, 0.9–2.8 h) before ^177^Lu-lilotomab satetraxetan injection.

**Table 1:** Patient characteristics. Median values (minimum to maximum) are indicated for continuous parameters

	Arm 1	Arm 2	Arm 3	Arm 4
10 MBq/kg dosage level (*n*)	2	1	1	0
15 MBq/kg dosage level (*n*)	3	2	3	1
20 MBq/kg dosage level (*n*)	2	0	0	4
Administered activity (MBq)	1435 (747–1982)	1137 (1013–1416)	1077.5 (891–1366)	1434 (1147–2189)
Sex (*n*, females)	1	1	3	0
Age (years)	53 (41–70)	71 (69–72)	75 (59–88)	72 (63–74)
Pre-dosage, lilotomab (mg)	40	0	0	199 (169–224)
Included for tumor dosimetry (*n*)	6	3	4	4
Included for RM dosimetry (*n*)	4	3	4	4
Included for biodistribution (*n*)	3	3	3	3

### Image acquisition

In brief, attenuation and scatter-corrected SPECT/CT images were acquired with a Siemens Symbia T16 scanner (Siemens Healthineers, Erlangen, Germany) at 96 and 168 h post injection (p.i.) of ^177^Lu-lilotomab satetraxetan. As from arm 2, an additional scan 24 h p.i. was performed. The acquisition covered areas of known lesions, and for at least three patients in each arm, the thorax and abdomen areas were also covered. Planar scintigraphy scans were acquired approximately 2, 24, 96, and 168 h p.i. The acquisition parameters and the methodological considerations have been described previously [8].

### Dosimetry

All patients with available imaging data were included for dosimetry. Absorbed doses to the tumors were calculated from SPECT/CT-images as described previously [7]. In brief, radioactivity in the lesions was obtained from the SPECT images at 96 and 168 h p.i. and time integrated activity coefficients were calculated using mono-exponential curve fitting. The SPECT/CT images obtained at 24 h p.i. were used to calculate time–activity curves from three time points for the lesions, however, to avoid systematic deviations, these results were only used for internal comparisons and not reported (as arm 1 patients were not imaged by SPECT/CT at 24 h p.i). The volumes were found from the CT images, and a distinct mass of minimum 1.5 ml volume was set as required for dosimetry to be performed. Patient mean tumor doses were found by averaging all available tumor absorbed doses for each patient.

RM absorbed dose was calculated with a SPECT/CT imaging-based approach using the activity in lumbar vertebrae 2–4 [6]. An imaging based method is here needed, as the assumption of equal radioactivity concentrations in blood samples and RM will underestimate the absorbed dose because of specific RM binding of the ARC [6, 9]. For two patients, one of the lumbar vertebrae was not covered. Homogenous uptake and equal mass of the three vertebrae were assumed and both activity and mass were multiplied with a correction factor of 1.5 for these two patients. Patients that had received prior external beam radiation therapy to the lumbar vertebrae were excluded.

Normal tissue absorbed doses for the remaining organs were calculated as previously described, defining spleen, liver, and kidneys as source organs [8]. The activities in these organs at different time points were primarily SPECT-derived. For time points where only planar imaging had been performed, the planar-derived organ counts were adjusted according to the ratio between the planar counts and SPECT activity values day 4. Individual masses were obtained from the CT images. For all other organs, the absorbed doses were calculated using OLINDA/EXM (version 1.1, Vanderbilt University, Nashville, TN, USA).

### Statistics

The patient mean tumor absorbed dose derived for each patient was divided by the RM self-dose for the patient to yield the tumor to RM ratio. Normal tissue absorbed doses, tumor absorbed dose, and the tumor to RM ratio were compared using a two-sided Student’s* t* test with a significance level of 0.05. In addition to the separate calculations, arms 2 and 3 were also combined and compared to arm 1 and 4 individually. The values for the combined group are hence obtained by averaging all absorbed dose values in the two arms (not by computing the mean value of the two arms’ mean values). The Shapiro–Wilk test and visual inspection of the quantile-quantile-plots showed that none of the data sets deviated substantially from a normal distribution. Thus, the parametric* t* test was used. The box plots show median values, interquartile ranges, the range of data indicated by whiskers, where points lower or higher than 1.5 times the lower or upper quartile displayed as outliers. All statistical calculations were conducted using Python version 2.7 (Python Software Foundation) with the SciPy (version 0.18) statistics library.

## Results

**Fig. 2:**
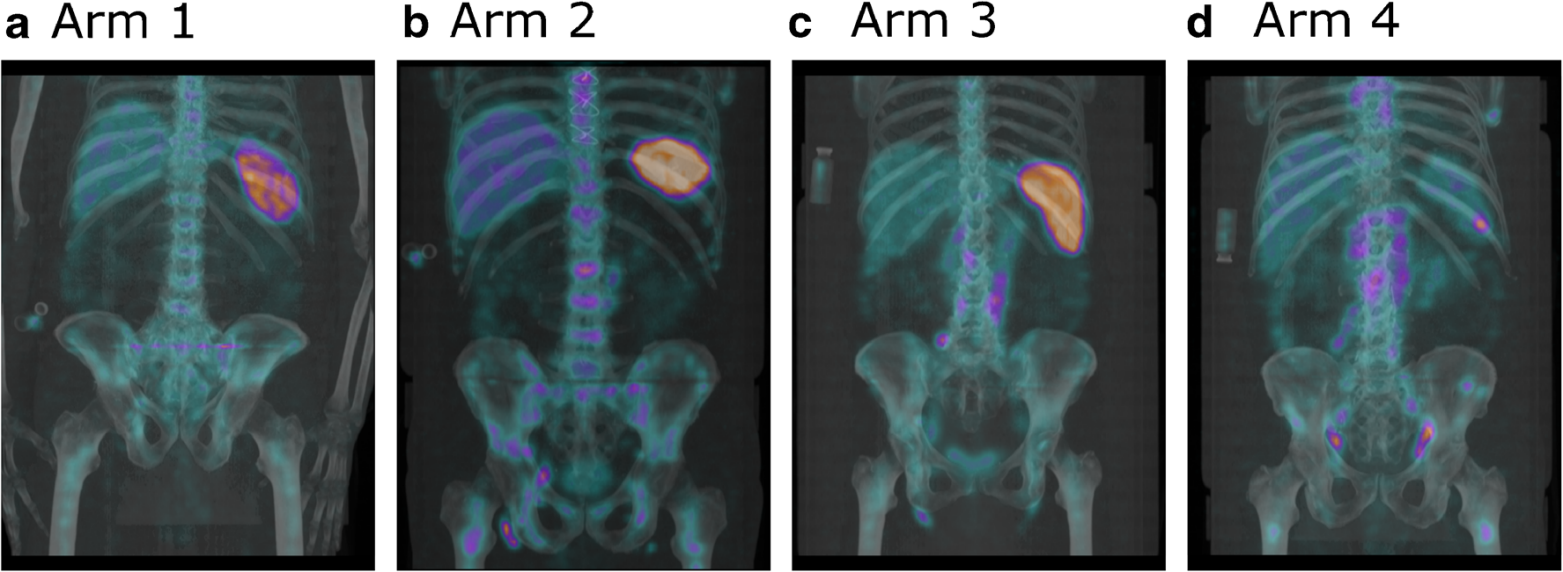
Fused SPECT/CT maximum intensity projection images at 96 h after injection of ^177^Lu-lilotomab satetraxetan.** a**–**d** Patients 009 (arm 1; 40 mg lilotomab), 013 (arm 2; no lilotomab), 017 (arm 3; no lilotomab), and 019 (arm 4; 100 mg lilotomab per m^2^ BSA) are shown. After 96 h, most of the ARC has been washed out of the blood, and ^177^Lu-lilotomab satetraxetan uptake is observed in tumors, liver, spleen, and bone marrow. A shift in uptake for the spleen can be observed when an increased amount of pre-dosing with lilotomab is given. Abdominal and inguinal lesions are visible, and the arm 4 patient also had a focal lesion in the spleen. All four patients had received 15 MBq/kg body weight of ^177^Lu-lilotomab satetraxetan, and the same intensity scale is used for all images

For ^177^Lu-lilotomab satetraxetan patients, the uptake was visually assessed to be most prominent in tumors, spleen, liver, and red marrow (Fig. 2). Also, radioactivity was seen in the blood (including heart cavities) at early time points.

### Tumor absorbed doses on a lesion level

**Fig. 3:**
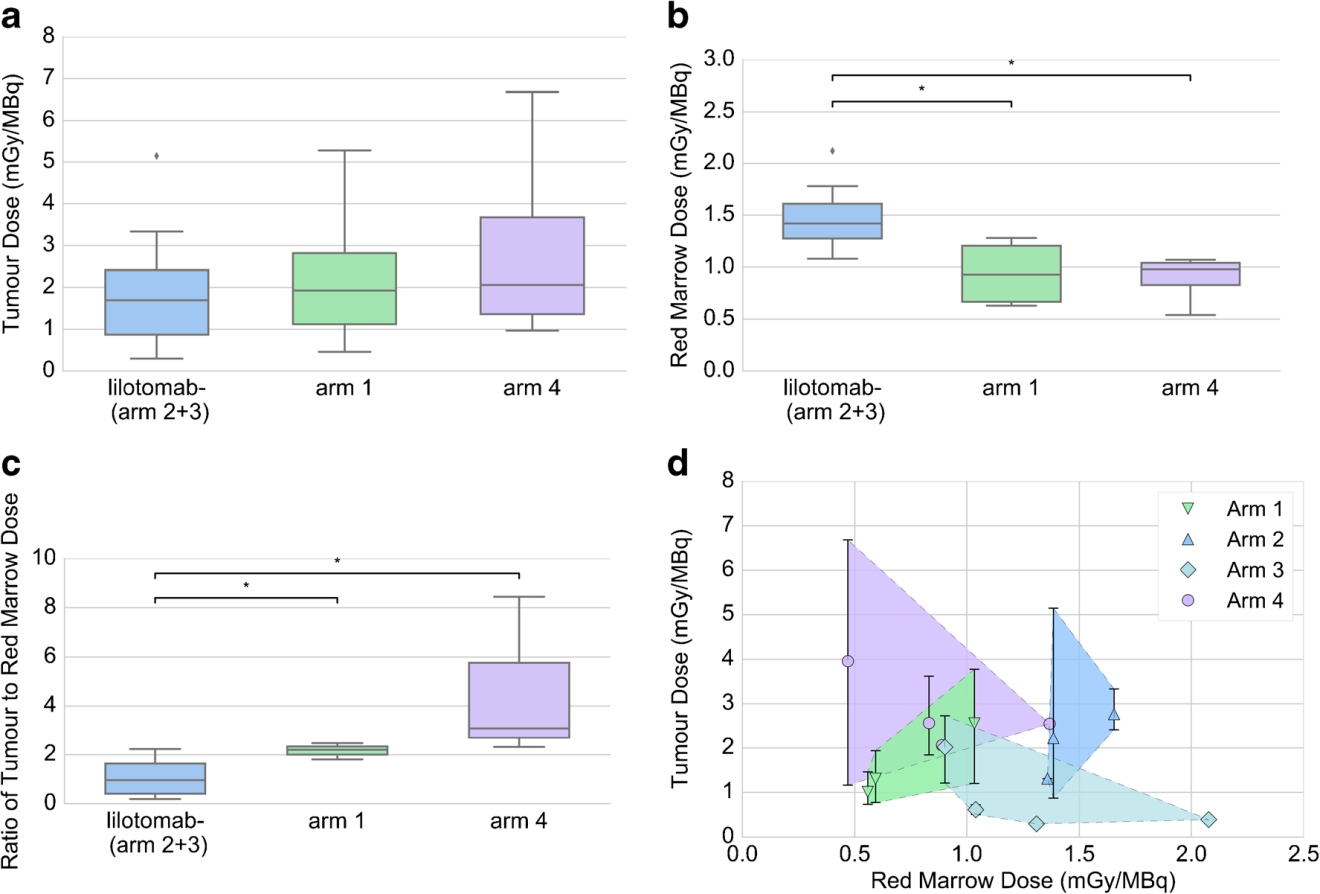
**a** The upper left boxplot displays tumor absorbed doses when different amounts of lilotomab are given as pre-dosing before ^177^Lu-lilotomab satetraxetan treatment. The three groups are obtained from patients that did not receive lilotomab (arms 2 and 3), patients that received 40 mg lilotomab (arm 1), and patients that received 100 mg lilotomab per m^2^ BSA (arm 4). The tumor absorbed doses are normalized by the amount of radioactivity given each patient. For all box plots, significant differences are annotated by asterisks. **b** Similar measures of the RM absorbed doses are also shown for the groups. Both arms 1 and 4 patients had received a significant lower RM absorbed dose than patients not pre-dosed with lilotomab. **c** The boxplot illustrates the ratios between patient mean tumor absorbed doses to RM absorbed doses. Here, the mean tumor absorbed dose is calculated for each patient before the patient ratios are obtained. Suppl. Fig. 1 displays the same box plots (**a**–**c**) separated for each of the four arms, as the rituximab timing differed between arms 2 and 3. **d** To illustrate the correlations between tumor and RM absorbed doses, the values are plotted against each other. The color coding separates the four arms. Each patient is represented by a symbol, and the patient mean tumor absorbed dose values are used (as in **c**). However, to show the variation in tumor absorbed doses, the intra-patient ranges are also displayed by a line interval between the maximum and minimum tumor absorbed doses for each patient. Some patients had only one lesion eligible for dosimetry, and therefore lack range indicators in panel** d**. The shaded areas are drawn using the extreme values from each arm

Seventeen of the 19 patients had one or more tumors eligible for dosimetry, and the number of lesions included per patient ranged from 1 to 5 (mode 3). A total of 47 tumor lesions were investigated, and tumor absorbed doses ranged from 33 to 859 cGy. For arms 1–4 the mean tumor absorbed doses per administered activity were 2.15, 2.31, 1.33, and 2.67 mGy/MBq, respectively. Combining arms 2 and 3, the mean tumor absorbed dose per administered activity for patients not pre-dosed with lilotomab was 1.79 mGy/MBq. No significant differences between tumor absorbed doses in this group and the individual arms with lilotomab pre-dosing (arm 1 and 4) were found. However, large variations in tumor absorbed dose were observed (Fig. 3a), and there were also found intra-patient variations with a range of up to 710 cGy (patient 019, arm 4) (Suppl. Table 1).

### Tumor absorbed doses on the patient level

Patient mean tumor absorbed doses per administered activity for each patient were calculated, and mean values for each arm were found to be 2.08, 2.10, 0.83, and 2.46 mGy/MBq for arms 1–4, respectively. Combining arms 2 and 3, the average patient mean tumor absorbed dose per administered activity was 1.37 mGy/MBq for the patient group not pre-dosed with lilotomab. Note that these values will differ slightly from the overall mean value per arm or group (given in the previous paragraph) since the absorbed doses are here averaged per patient before the mean is obtained. There was a slightly higher patient mean tumor absorbed dose in arm 4 patients compared to the non-pre-dosed group, but the difference was not significant (*p* = 0.13).

### RM absorbed doses

Fifteen patients were included for RM dosimetry, as two of the patients had received prior external beam radiation therapy to the lumbar vertebrae and two of the patients lacked imaging data of the area. The RM absorbed doses ranged from 69 to 204 cGy. Mean RM absorbed doses for the different arms were 0.94, 1.55, 1.44, and 0.89 mGy/MBq for arms 1–4, respectively. Mean absorbed dose for the arms that were not pre-dosed with lilotomab was 1.48 mGy/MBq. There was a significantly higher RM absorbed dose in this non-pre-dosed group compared to arm 1 (*p* = 0.04) and arm 4 (*p* = 0.02) (Fig. 3b).

### Absorbed doses for the rest of normal tissues

The mean spleen absorbed doses were 2.81, 3.12, 3.27, and 1.13 mGy/MBq for arms 1–4, respectively. Combining arm 2 and 3, the mean dose was 3.20 mGy/MBq for the group not pre-dosed with lilotomab. There was a significantly lower spleen dose in arm 4 patients compared to this group. No significant differences in absorbed doses between each arm were found for the rest of the normal tissues (Table 2). Individual masses, time-integrated activity coefficients, and absorbed doses for all patients can found in supplementary Table 1.

**Table 2:** Absorbed doses to all organs for the different pre-dosing and pre-treatment regimens

	Arm 1	Arm 2	Arm 3	Arm 4
	Mean (range) mGy/MBq	Mean (range) mGy/MBq	Mean (range) mGy/MBq	Mean (range) mGy/MBq
Adrenals	0.12 (0.10–0.14)	0.10 (0.07–0.12)	0.11 (0.07–0.17)	0.12 (0.11–0.13)
Brain	0.10 (0.08–0.13)	0.08 (0.05–0.10)	0.09 (0.05–0.15)	0.10 (0.10–0.11)
Breasts	0.10 (0.08–0.12)	0.08 (0.05–0.10)	0.09 (0.05–0.15)	0.10 (0.09–0.11)
Gallbladder wall	0.12 (0.10–0.14)	0.10 (0.07–0.12)	0.11 (0.07–0.17)	0.12 (0.12–0.14)
LLI wall	0.11 (0.09–0.13)	0.09 (0.06–0.11)	0.09 (0.06–0.16)	0.11 (0.10–0.12)
Small intestine	0.11 (0.09–0.13)	0.09 (0.06–0.11)	0.10 (0.06–0.16)	0.11 (0.10–0.12)
Stomach wall	0.11 (0.09–0.13)	0.09 (0.06–0.11)	0.10 (0.07–0.16)	0.11 (0.10–0.12)
ULI wall	0.11 (0.09–0.14)	0.09 (0.06–0.11)	0.10 (0.06–0.16)	0.11 (0.10–0.12)
Heart wall	0.11 (0.09–0.13)	0.09 (0.06–0.11)	0.10 (0.06–0.16)	0.11 (0.10–0.12)
Kidneys	0.46 (0.28–0.79)	0.25 (0.16–0.30)	0.38 (0.30–0.47)	0.49 (0.34–0.71)
Liver	0.97 (0.74–1.15)	0.95 (0.78–1.05)	1.02 (0.70–1.43)	0.96 (0.69–1.30)
Lungs	0.11 (0.09–0.13)	0.09 (0.06–0.11)	0.09 (0.06–0.16)	0.11 (0.10–0.12)
Muscle	0.10 (0.08–0.13)	0.08 (0.06–0.10)	0.09 (0.06–0.15)	0.10 (0.10–0.11)
Ovaries	0.11 (0.09–0.13)	0.09 (0.06–0.11)	0.09 (0.06–0.16)	0.11 (0.10–0.12)
Pancreas	0.12 (0.1–0.14)	0.10 (0.07–0.12)	0.11 (0.08–0.18)	0.12 (0.11–0.14)
Red marrow	0.94 (0.63–1.28)	1.55 (1.42–1.78)	1.44 (1.08–2.12)	0.89 (0.54–1.07)
Osteogenic cells	0.50 (0.31–0.70)	0.87 (0.73–1.03)	0.80 (0.63–0.91)	0.56 (0.30–0.74)
Skin	0.10 (0.08–0.12)	0.08 (0.05–0.09)	0.08 (0.05–0.14)	0.10 (0.09–0.11)
Spleen	2.81 (1.54–3.60)	3.12 (2.73–3.45)	3.27 (2.65–4.01)	1.13 (0.78–1.43)
Testes	0.10 (0.08–0.13)	0.08 (0.05–0.10)	NA	0.10 (0.10–0.11)
Thymus	0.11 (0.09–0.13)	0.08 (0.06–0.10)	0.09 (0.06–0.15)	0.10 (0.10–0.11)
Thyroid	0.10 (0.08–0.13)	0.08 (0.05–0.10)	0.09 (0.05–0.15)	0.10 (0.10–0.11)
Urinary bladder wall	0.11 (0.09–0.13)	0.08 (0.06–0.10)	0.09 (0.06–0.15)	0.10 (0.10–0.11)
Uterus	0.11 (0.09–0.13)	0.09 (0.06–0.11)	0.09 (0.06–0.16)	0.11 (0.10–0.12)
Total body	0.14 (0.12–0.17)	0.13 (0.11–0.15)	0.14 (0.08–0.20)	0.15 (0.11–0.19)

### Ratios between tumor and RM absorbed dose

The tumor to RM absorbed dose ratios were calculated using the patient mean tumor absorbed doses, and the mean ratios were 1.07, 2.16, and 4.62 for patients not pre-dosed with lilotomab, arm 1, and arm 4, respectively (Fig. 3c). There was a significantly higher tumor to RM absorbed dose in both arm 1 (*p* = 0.05) and arm 4 (*p* = 0.02) compared to the non-pre-dosed group. The ratios calculated separately for arms 2 and 3 gave values of 1.41 and 0.81 (Suppl. Fig. 1).

## Discussion

In this study, we have investigated normal tissue and tumor absorbed doses for ^177^Lu-lilotomab satetraxetan therapy following four different pre-treatment and pre-dosing regimens. For all four arms, RM was found the primary dose-limiting organ, and both pre-dosing amounts with lilotomab investigated had a mitigating effect on RM absorbed dose. Increasing the amount of lilotomab did reduce the RM and spleen absorbed doses, however, the decrease was not significant. The ratio of tumor to RM absorbed dose was found to significantly increase for both the patient group given 40 mg of lilotomab and the group given 100 mg/m^2^ of lilotomab compared to patients not pre-dosed with lilotomab.

In our previous work, we have shown that the RM absorbed dose decreased when 40 mg of lilotomab was given as pre-dosing before ^177^Lu-lilotomab satetraxetan therapy [6]. In the current study, the approximately fivefold increase in the amount of lilotomab that was given to patients in arm 4 was found to cause a further minor decrease in RM absorbed dose. Only four patients were included from each of the two arms and a larger number of patients is required for statistical verification of this trend. Still, a lower RM absorbed dose is also supported by that a higher radioactivity level could be given to patients in arm 4 (Fig. 1). There is a concern that pre-dosing with cold antibody could block the CD37 antigen on tumor tissues as well, but on a lesion level there was no significant difference in the tumor absorbed dose for arm 1, arm 4, and not pre-dosed patients. However, the overall variation in tumor absorbed doses was highest in arm 4, so we cannot exclude that uptake was influenced by the lilotomab pre-dosing for some lesions (Fig. 3a).

The amount of unlabeled antibody that produced the highest ratio of tumor to whole-body absorbed dose was investigated for individual patients in the first ^131^I-tositumomab trials [10]. In the current work, we calculated the ratio of tumor to RM absorbed dose to compare all ^177^Lu-lilotomab satetraxetan therapy regimens. The ratio doubled for patients receiving 40 mg lilotomab and doubled again for the patients given 100 mg/m^2^ BSA of lilotomab, indicating that the higher pre-dosing level can optimize the therapeutic effect. It should, however, be noted that the ratio parameter considers the mean tumor absorbed dose across all lesions per patient, and large intra-patient variations in tumor absorbed dose can possibly be obscured. The variation is visualized in Fig. 3d. The limited number of patients in each group with more than two tumors eligible for dosimetry does not allow for statistical comparisons, but for arm 4 the intra-patient tumor absorbed doses variation appears somewhat larger, and the largest intra-patient range (710 cGy) was found in this arm (Suppl. Table 1). Here, we aimed to perform an overall assessment of the different groups rather then effect prediction for individual patients. For such prediction studies, a more suiting parameter could perhaps be the ratio of the patient *minimum* tumor absorbed dose to RM absorbed dose. However, the same trend is shown using this parameter; increasing values for non-pre-dosed patients, arm 1, and arm 4 (data not shown). A larger uncertainty will possibly be introduced by such a parameter, since not all lesions are eligible for dosimetry.

There were some differences in absorbed dose for other normal tissues, but the only significant difference was for the spleen. This absorbed dose was significantly lower for arm 4 patients than for patients not pre-dosed with lilotomab (Table 2). All organs received absorbed doses within commonly assumed tolerance levels, e.g., the highest spleen absorbed dose across all patients was 6.5 Gy (patient 005), which is lower than the absorbed doses observed to have an effect for other lutetium-177-based treatments [11, 12]. Accordingly, no signs of non-hematological toxicities were observed for the included patients. For the calculation of ratios between tumors and organs-at-risk, we therefore focused on RM as the most important normal tissue.

Pre-dosing with unlabelled antibody as a means of improving biodistribution has been demonstrated effective for ARCs targeting CD20. Treatment with ^131^I-tositumomab was described to be preceded by 450 mg cold tositumomab [13] and ^90^Y-ibritumomab tiuxetan treatment uses 250 mg/m^2^ BSA cold anti-CD20 rituximab as pre-dosing [14]. The theory is that unlabeled antibody will bind the circulating non-malignant B cells expressing target antigens. Administration of pre-dosing therefore prevents rapid ARC sequestration in the spleen and will, as a result, prolong the ARCs’ plasma half-life [15]. Reduction of ARC uptake in the spleen is clearly visible, for example ^131^I-tositumomab [13], and while the spleen uptake even without lilotomab pre-dosing was lower for ^177^Lu-lilotomab satetraxetan, a corresponding reduction can also be observed for this treatment (Fig. 2). We have found that the cumulative activity in blood was higher, and the clearance was lower, for arm 1 patients than for arm 2 patients [6]. The higher amounts of lilotomab given patients in arm 4 increased the cumulative radioactivity in blood even further [16]. This may also explain the somewhat higher mean tumor absorbed dose in arm 4; if the concentration in the blood increases, this may lead to overcoming the binding site barrier and increase diffusion into the tumor [17]. The observed changes in biodistribution may indicate that the same mechanics are applicable for pre-dosing with lilotomab before ^177^Lu-lilotomab satetraxetan treatment as for previous ARC pre-dosing regimens. For the patients enrolled so far, the maximum amount of lilotomab pre-dosing has been 224 mg (Table 1). This is lower than the pre-dosing amounts given for ^131^I-tositumomab or ^90^Y-ibritumomab tiuxetan, but can be considered in relative agreement with that the antigen expression of CD37 has been measured approximately half of the CD20 expression in vitro [5, 18]. It is uncertain whether lilotomab pre-dosing levels above 100 mg/m^2^ BSA could prove beneficial. While the increased tumor to RM ratios encourage such investigations, the larger absorbed dose variation for lesions may advise against a continued escalation of pre-dosing amounts. In studies of an iodine-131 labeled anti-CD37 antibody, MB-1, three different protein amounts were investigated, 0.5, 2.5, and 10 mg/kg, which corresponds to 35, 175, and 700 mg for a 70 kg patient [19, 20]. The highest amount yielded the most favorable biodistribution in the majority of patients. Interestingly, the cold antibody was given simultaneously as the ARC (not as pre-dosing), and one should then believe that both cold and radiolabeled antibodies would bind non-malignant B cells and tumors with the same relative effect. This difference in administration does make direct comparisons with the ^177^Lu-lilotomab satetraxetan pre-dosing regimens challenging. However, this calls for a closer investigation of the amount of radiolabeled antibody given. For arm 1, the amount of ^177^Lu-lilotomab satetraxetan was relatively invariable, and for arm 4 the amount was less than 5% of the amount given as cold lilotomab pre-dosing. A clear deviation was found for one of the patients that did not receive lilotomab pre-dosing, as this patient was given approximately twice the amount of radiolabeled antibody compared to the rest (patient 18, 16.4 mg, supplementary Table 1). While we cannot rule out that variable amounts of radiolabeled antibody will impact the biodistribution, the RM and tumor absorbed doses for patient 18 were within the range of the other arm 3 patients. The absolute amounts of radiolabeled CD37 antibody given are probably too low for any measurable effects of possible differences.

Two arms excluding lilotomab as pre-dosing have been investigated. In the present work, the absorbed doses have been reported separately for these two arms, since the rituximab timing varied between the arms. However, no large differences were observed (Table 2), and the data were combined for some of the analyses and discussion. Pre-dosing with the anti-CD20 targeting rituximab on the same day as ^177^Lu-lilotomab satetraxetan was investigated in arm 3 because rituximab will also bind Fcγ receptors [21], and although lilotomab is a mouse antibody, it also binds to subtypes of human Fcγ receptors. Our results show that the rituximab pre-dosing will not introduce the same protective effect for RM as pre-dosing with the same anti-CD37 antibody as is part of the ARC, indicating that the blocking mechanics discussed above are antibody-specific. This is in accordance with that rituximab has been found to block radiolabeled anti-CD20 antibodies, but not radiolabeled anti-CD45 antibodies [22].

In general, biodistribution and dosimetry studies allow for the determination of uptake in organs-at-risk and tumors, and hence the selection of an optimal pre-treatment and pre-dosing regimen. It is an open question as to whether this process should be conducted before activity level escalation (often called dose-escalation) is performed. This could minimize the number of patients in arms that are later judged less effective. While the correlation of RM absorbed dose and hematological toxicity has been demonstrated for the current phase 1/2a trial [6], the observed variation in tumor absorbed dose prevents fully reliable clinical translation, and further studies are needed to investigate tumor absorbed dose vs. patient outcome. If this issue is resolved, a tumor to RM absorbed dose ratio parameter could prove valuable for predictive purposes.

## Conclusions

For all patient arms, RM was found to be the primary dose-limiting organ for ^177^Lu-lilotomab satetraxetan therapy, and pre-dosing with lilotomab had a mitigating effect on RM absorbed dose. Increasing the amount of lilotomab was found to reduce the RM absorbed dose, and the ratio of tumor to RM absorbed dose was found to double. Still, based on the dosimetry data, the variation in tumor absorbed doses leaves the question of the optimal amount of lilotomab somewhat inconclusive. Continued investigations of absorbed dose–effect correlations are therefore needed. However, as both pre-dosage levels investigated significantly increased the tumor to RM absorbed dose ratio, it seems mandatory to include pre-dosing with lilotomab in a treatment regimen with ^177^Lu-lilotomab satetraxetan.

## Electronic supplementary material

Supplementary Table 1 (PDF 147 kb)

Supplementary Fig. 1 (PDF 159 kb)
