# Supplementary material for: Pre-dosing with lilotomab prior to therapy with 177Lu-lilotomab satetraxetan significantly increases the ratio of tumor to red marrow absorbed dose in non-Hodgkin lymphoma patients
Source: Eur J Nucl Med Mol Imaging. 2018 Feb 22;45(7):1233–41. doi: 10.1007/s00259-018-3964-9 (PMC5953993; doi:10.1007/s00259-018-3964-9)
Supplement: Supplementary file 1 — (PDF 147 kb) [file 259_2018_3964_MOESM1_ESM.pdf]

SUPPLEMENTARY TABLE 1.

Individual patient characteristics, as well as absorbed doses, masses and time-integrated activity coefficients (TIACs) for the target organs.

“N.A.” indicates that insufficient imaging data are available, that the red bone marrow (RM) has been treated by external beam radiation previously, or that no tumours were eligible for dosimetry.

Patient number	Sex	Arm	Prescription level [MBq/kg]	Body weight [kg]	Injected activity [MBq]	Amount of labelled lilotomab [mg]	Amount of lilotomab pre-dosing [mg]	Liver			Spleen			Kidney			RM	Tumours Mean (range)
								Mass (g)	TIAC (h)	Dose (Gy)	Mass (g)	TIAC (h)	Dose (Gy)	Mass (g)	TIAC (h)	Dose (Gy)	Dose (Gy)	Dose (Gy)
1	F	3	10,0	118,0	1102,0	N.A.	0,0	1876,0	14,6	0,8	262,0	8,0	2,9	204,0	0,7	0,3	1,5	0.33 (0.33-0.33)
2	M	1	10,0	103,0	1036,0	7,9	40,0	2405,0	19,8	0,8	406,0	7,2	1,6	233,0	0,7	0,3	0,7	1.04 (0.76-1.51)
3	M	1	10,0	73,0	746,0	7,9	40,0	1295,0	16,7	0,9	100,0	4,2	2,7	117,0	1,1	0,6	0,9	1.91 (0.89-2.82)
5	M	1	20,0	98,0	1982,0	8,3	40,0	1705,0	19,4	2,0	194,0	7,3	6,5	270,0	0,9	0,6	N.A.	2.09 (0.91-3.28)
7	M	1	20,0	74,0	1505,0	8,1	40,0	N.A.	N.A.	N.A.	N.A.	N.A.	N.A.	N.A.	N.A.	N.A.	N.A.	5.06 (3.19-7.94)
9	M	1	15,0	110,0	1696,0	7,8	40,0	N.A.	N.A.	N.A.	N.A.	N.A.	N.A.	N.A.	N.A.	N.A.	1,2	2.21 (1.31-3.28)
11	M	1	15,0	97,0	1435,0	8,9	40,0	N.A.	N.A.	N.A.	N.A.	N.A.	N.A.	N.A.	N.A.	N.A.	N.A.	4.57 (2.77-6.72)
12	F	1	15,0	67,0	1015,0	8,9	40,0	N.A.	N.A.	N.A.	N.A.	N.A.	N.A.	N.A.	N.A.	N.A.	1,3	N.A
13	M	2	15,0	94,0	1416,0	9,5	0,0	1690,0	20,0	1,5	170,0	6,8	4,9	211,0	0,7	0,4	2,0	3.15 (1.23-7.28)
14	F	2	15,0	65,0	1013,0	9,1	0,0	1687,0	19,1	1,0	152,0	5,6	3,2	147,0	0,5	0,3	1,8	2.80 (2.45-3.38)
15	M	2	10,0	112,0	1137,0	8,8	0,0	2030,0	17,6	0,9	187,0	5,9	3,1	282,0	0,5	0,2	1,6	1.48 (1.48-1.48)

16	F	3	15,0	59,0	891,0	8,2	0,0	1290,6	20,8	1,3	214,9	10,0	3,6	120,1	0,6	0,4	1,9	0.35 (0.35-0.35)
17	F	3	15,0	69,0	1053,0	8,3	0,0	1644,2	16,9	1,0	260,4	9,4	3,3	171,7	0,7	0,4	1,1	2.12 (1.27-2.87)
18	M	3	15,0	92,0	1366,0	16,4	0,0	N.A.	N.A.	N.A.	N.A.	N.A.	N.A.	N.A.	N.A.	N.A.	1,6	0.84 (0.69-0.98)
19	M	4	15,0	85,0	1286,0	8,6	199	N.A.	N.A.	N.A.	N.A.	N.A.	N.A.	N.A.	N.A.	N.A.	0,7	5.09 (1.49-8.59)
21	M	4	20,0	91,0	1769,0	5,4	207	2818,8	21,6	1,2	398,7	3,5	1,4	161,9	0,8	0,7	1,6	4.59 (3.30-6.49)
22	M	4	20,0	59,0	1147,0	3,4	169	N.A.	N.A.	N.A.	N.A.	N.A.	N.A.	N.A.	N.A.	N.A.	1,2	2.36 (2.36-2.36)
23	M	4	20,0	72,0	1434,0	4,3	191	2105,4	30,6	1,9	566,6	9,3	2,1	153,6	1,3	1,0	1,5	N.A
25	M	4	20,0	109,0	2189,0	6,3	224	1790,4	17,7	1,9	198,7	2,7	2,6	219,6	0,8	0,8	N.A.	2.67 (2.12-3.43)
