# Supplementary material for: Pre-dosing with lilotomab prior to therapy with 177Lu-lilotomab satetraxetan significantly increases the ratio of tumor to red marrow absorbed dose in non-Hodgkin lymphoma patients
Source: Eur J Nucl Med Mol Imaging. 2018 Feb 22;45(7):1233–41. doi: 10.1007/s00259-018-3964-9 (PMC5953993; doi:10.1007/s00259-018-3964-9)
Supplement: Supplementary file 2 — (PDF 159 kb) [file 259_2018_3964_MOESM2_ESM.pdf]

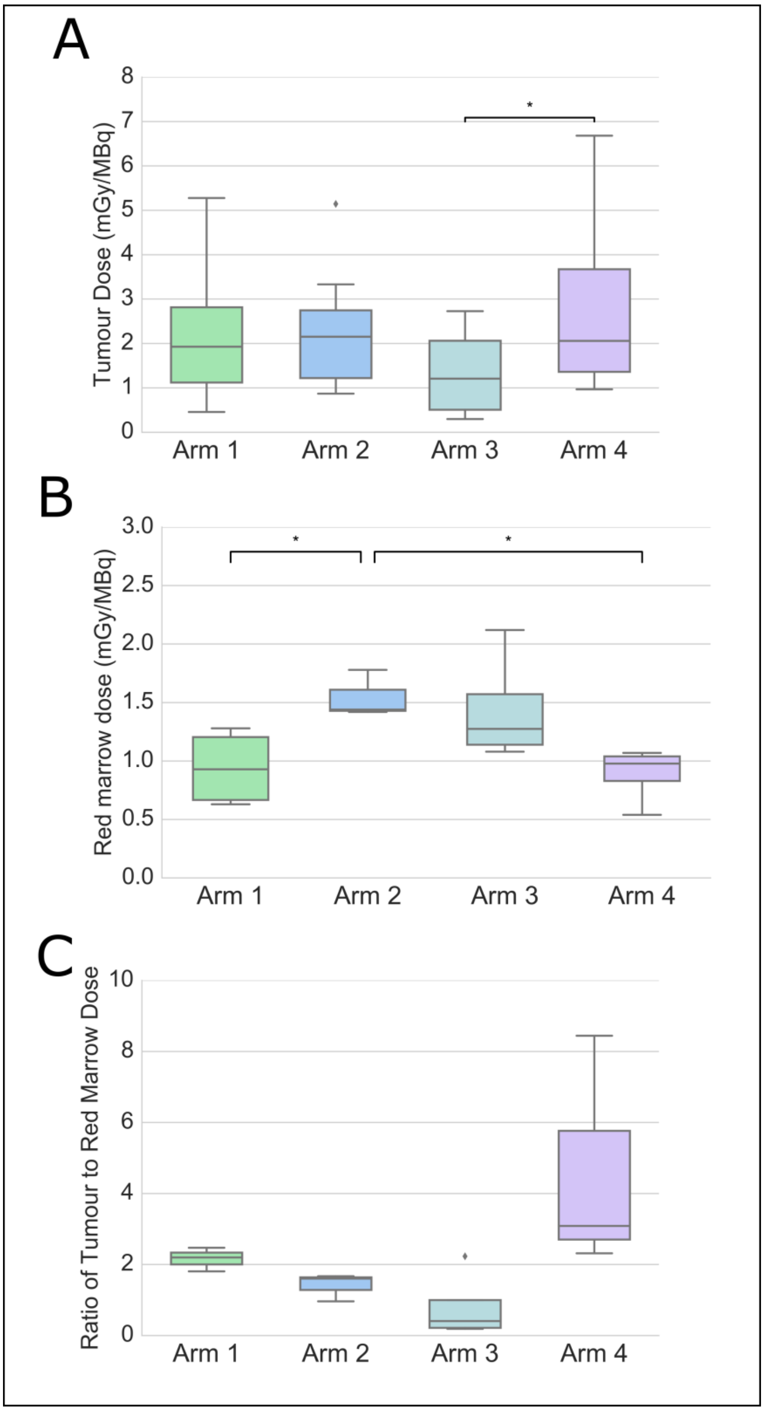

Supplementary Figure 1. (A) The upper boxplot displays tumour absorbed doses when different amounts of lilotomab are given as pre-dosing before ^{177}Lu-lilotomab satetraxetan treatment. The four patient arms are shown. Arms 2 and 3 did not receive lilotomab, arm 1 patients received 40 mg lilotomab and arm 4 patients received 100 mg lilotomab per m^2 BSA. The tumour absorbed doses are corrected by the amount of radioactivity given each patient. For all box plots, significant differences are annotated by asterisks. (B) Similar measures of the RM absorbed doses are also shown for the groups. (C) The boxplot illustrates the ratios between patient mean tumour doses to RM absorbed doses. Here, the mean tumour absorbed dose is calculated for each patient before the patient ratios are obtained. Fig. 1 displays the same box plots when arms 2 and 3 are grouped.
